# Early Postoperative Functional Recovery in Older Patients With Periprosthetic Femoral Fractures: Comparison Between Cemented and Cementless Stem Revisions

**DOI:** 10.1016/j.artd.2024.101467

**Published:** 2024-07-20

**Authors:** Hideki Ueyama, Mitsuyoshi Yamamura, Junichiro Koyanagi, Kenji Fukunaga, Susumu Takemura, Suguru Nakamura

**Affiliations:** a Department of Orthopedic Surgery, Osaka Rosai Hospital, Sakaishi, Osaka, Japan; b Department of Orthopedic Surgery, Sano Memorial Hospital, Izumisanoshi, Osaka, Japan

**Keywords:** Periprosthetic femoral fracture, Unified classification system type B2, Cemented stem revision, Functional recovery, Older patients

## Abstract

**Background:**

Early postoperative functional recovery is important in older patients with lower-extremity fractures to prevent disuse, and periprosthetic femoral fractures (PFFs) are no exception. This study aimed to compare the postoperative functional recovery in the early phase after revision for PFF with loose stems between cemented and cementless stems.

**Methods:**

Eighteen patients with Unified Classification System type B2 PFF were included in this retrospective cohort study with a follow-up period of about 2 years. All patients underwent stem revision and were divided into 2 groups: the cemented stem group (n = 9) and the cementless stem group (n = 9). In postrevision, functional independence measure score, independent walk rate, activities of daily living recovery rate to the original level at 2 weeks postoperatively, the Beals and Tower classification for radiological status, and survival rate for readmission as endpoints were compared between the 2 groups.

**Results:**

Patients in the cemented group recovered functional mobility earlier than in the cementless group, with higher postoperative functional independence measure functional subscale values (73 vs 50 points, *P* = .02), higher independent walk rate (89 vs 11%, *P* < .01), and more postoperative activities of daily living recovery (100% vs 44%, *P* = .03) at 2 weeks postoperatively. The Beals and Tower classification and survival rates were similar in both groups.

**Conclusions:**

Revision using a cemented stem for PFF in older patients was a useful surgical procedure in terms of early postoperative functional recovery. Cemented stem revision was comparable with cementless in bone union and safety at 2 years postoperatively.

## Introduction

Life expectancy has increased worldwide, although there are differences based on ethnicity and region [1]. The number of patients undergoing total hip arthroplasty (THA) is increasing, and older patients are expected to become eligible and live longer thereafter [2].

Periprosthetic fractures are a complication of THA. Periprosthetic femoral fractures (PFFs) are more common than pelvic fractures [3], and older age has been reported as a risk factor for PFF [4]. As the number of post-THA patients increases and age increases, PFF is speculated to be more frequent; therefore, it is important to optimize the treatment of PFF in the older patients.

Research has demonstrated that older patients experience weakened skeletal muscle strength when mobility is restricted, even for short durations, such as 1 week of bed rest [5]. Lower extremity muscle weakness in older patients leads to disadvantages such as increased risk of falls, readmission rate, and cost of care [6]. Among patients with hip fractures, those who initiated walking within 48 hours postsurgery exhibited early functional recovery and reduced dependency on additional medical care compared to those who delayed walking [7]. Therefore, it is important for patients with PFF to walk in the early postoperative phase.

Although internal fixation can be an appropriate treatment option if stable fixation can be achieved, the standard treatment for Unified Classification System (UCS) type B2 PFF [8], a fracture type with stem loosening and without bone defects, is revision with a long stem to bypass the fracture site [9,10]. However, few reports have compared the results of revision using cemented and cementless stems for UCS type B2 PFFs. Although there is one study in which the primary outcome was mid-term postoperative survivorship [11], no studies have compared the 2 surgical procedures in terms of early postoperative functional recovery. Reports suggest that hip arthroplasty with cemented stems offers advantages, including improved early loading tolerance and pain reduction, compared to cementless stems [12]. Therefore, we hypothesized that cemented stem revision would have a favorable effect on early functional recovery even in older patients with UCS type B2 PFFs. However, a comparison of the results regarding early functional recovery between cemented and cementless stems in PFF was unknown. This study aimed to compare early postoperative functional recovery after revision using cemented and cementless stems in older patients with UCS type B2 PFFs.

## Material and methods

### Study design

**Figure 1:**
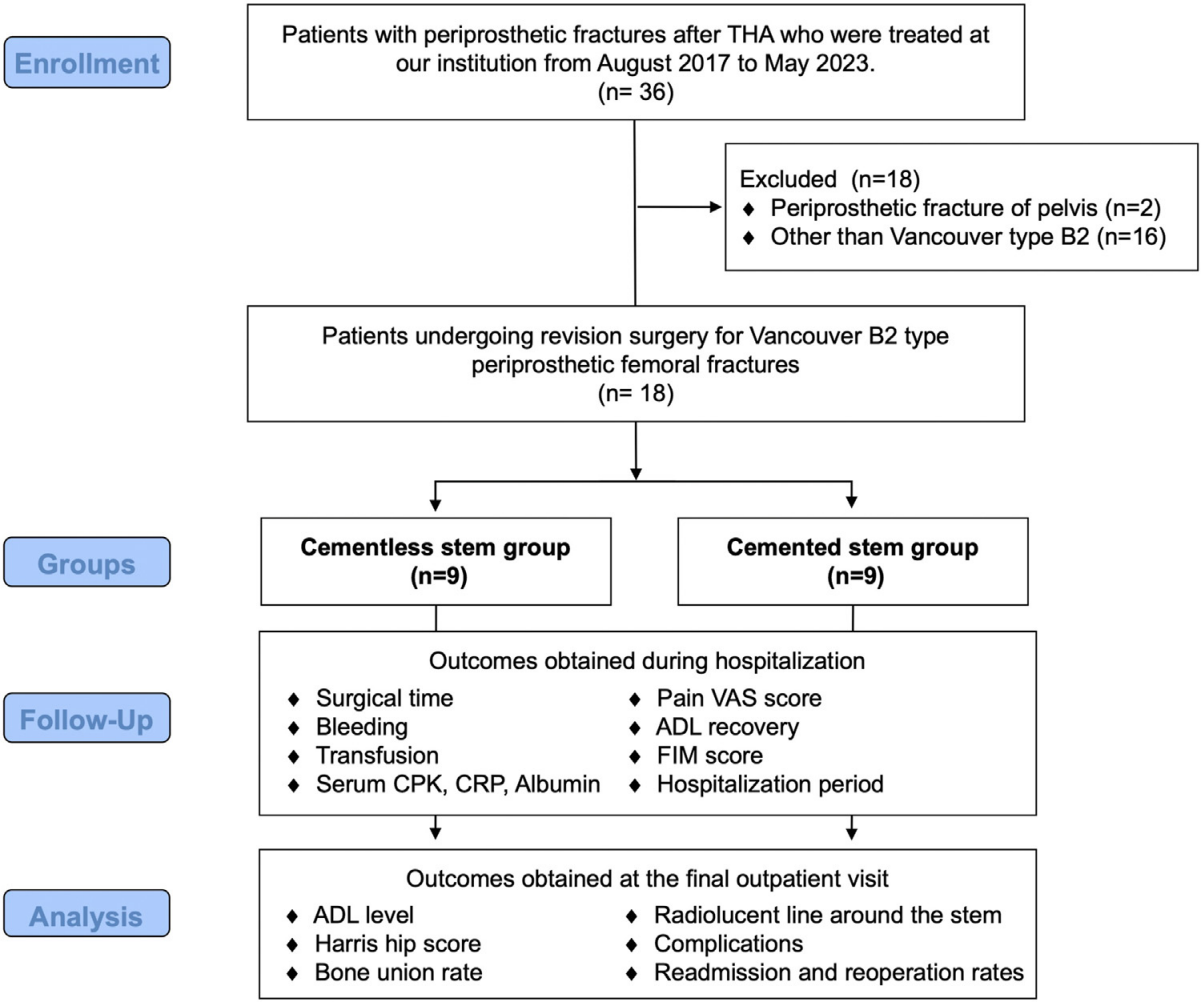
Flowchart of this study. This retrospective cohort study compared the early postoperative functional recovery after revision surgery for Unified Classification System type B2 periprosthetic femoral fractures between cemented and cementless stems.

This retrospective cohort study included patients with periprosthetic fractures after THA who were treated at our institution between August 2017 and May 2023 (Fig. 1). The inclusion criteria were patients undergoing revision for UCS type B2 PFF. Eighteen patients were included in this study. This study was approved by the institutional review board (approval number 2023-31). All patients provided informed consent prior to enrolling in the study.

### Surgical procedures and postoperative management

**Figure 2:**
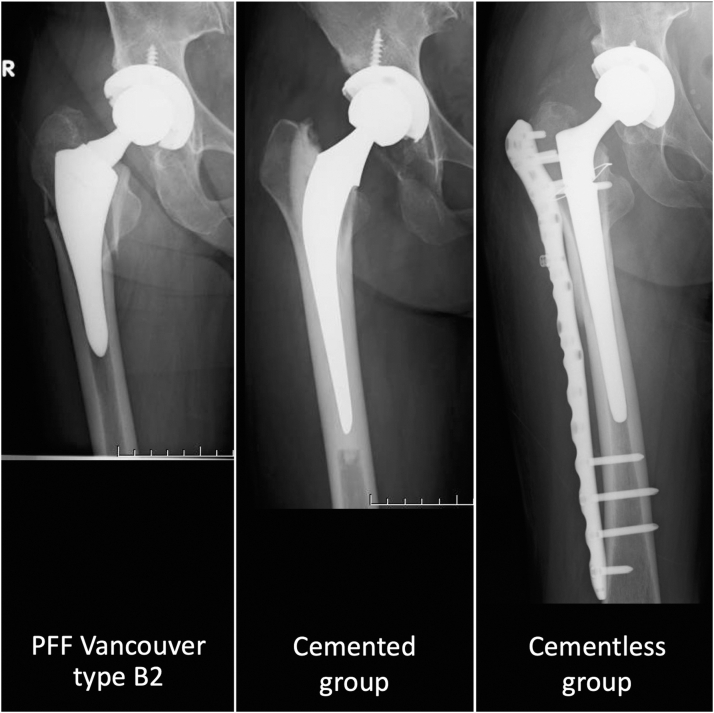
Representative radiographs of revision surgeries. A femoral fracture and sinking of the stem were observed and diagnosed as Vancouver type B2 periprosthetic femoral fracture (left picture). Representative radiographs of revision surgery with a cemented stem (center picture) and a cementless stem (right picture).

PFF were diagnosed by the surgeon in each case using radiographs and computed tomography according to the UCS classification [8]. All patients with UCS type B2 PFF underwent revision using a long stem to bypass the femoral fracture site. Cementless stems were used in all patients before March 2020 (cementless group), and cemented stems were used in the other patients (cemented group) (Fig. 2). Nine patients were in the cemented group, and the other 9 were in the cementless group. The cementless stem was cone-shaped with fins for the distal fixed stem (Modulus R; Lima Corporate, Villanova di San Daniele del Friuli, Italy), and a locking plate was used for fracture fixation (NCB plate; ZimmerBiomet, Warsaw, IN). The cemented stem was of a composite beam type (CMK or Versys Advocate, ZimmerBiomet, Warsaw, IN), and a cable system was used for fracture fixation (NESPLON cable system, Alfresa Pharma Corporation, Tokyo, Japan). In all cases in the cemented group, cables were used for fracture fixation, and plates were not used. This 2-group comparison was a historical control comparison [13]. Postoperative therapy in both groups was performed by physiotherapists. Patients were allowed to walk with support, even if they could not fully weight-bear. In the cementless group, weight-bearing at the surgical site was limited for the first 4-6 weeks after surgery to prevent fracture displacement, whereas full loading was allowed immediately after surgery in the cemented group. Pain and nutritional management during hospitalization were identical in both groups, according to the institutional protocol. The time of discharge was determined by the surgeon when the patient was out of the acute phase of postoperative care, according to the individual circumstances.

### Outcomes

The outcomes were as follows: *surgical parameters*, surgical time, blood loss, transfusion rate, serum creatine phosphokinase (CPK), serum C-reactive protein, change in serum albumin, *clinical parameters*, Harris hip score, pain visual analog scale (VAS), functional independence measure (FIM), activities of daily living (ADL) recovery, the independent walk rate, readmission rate, reoperation rate, hospitalization period, *radiological parameters*, Beals and Tower classification, and radiolucent line around the stem. The details of each outcome are as follows: Surgical time was defined as the time from the start of the surgery to skin closure. Blood loss was defined as the mass of bleeding aspirated by the suction instrument at the surgical site and wiped away with gauze during surgery. The transfusion rate was recorded during hospitalization. The transfusion rate was calculated based on whether blood was transfused during hospitalization. The decision to transfuse was made by the surgeon when the patient had a hemoglobin below 65 g/L, the institutional standard for transfusion, and was symptomatic. Serum CPK and C-reactive protein levels were defined as the highest values obtained in blood tests performed at 1, 4, 7, and 14 days postoperatively. The change in serum albumin level was defined as the difference between the highest and lowest values during the same period. The Harris hip score was measured before primary THA, described as the initial Harris hip score, and at final observation after PFF surgery [14]. Pain VAS scores were recorded by a nurse on days 1, 4, 7, 14, and 21 postoperatively on a 10-point scale, with 0 indicating no pain and 10 indicating the worst pain. The minimal clinically important difference in the pain VAS was defined as 1.86 based on a previous report [15]. Differences in the pain VAS between preoperative and 2 weeks postoperatively were assessed. FIM was assessed by a physiotherapist, and functional (13-91 points) and mental (5-35 points) subscale scores were recorded, with higher scores indicating better ADL [16,17]. FIM was assessed before primary THA, the initial FIM, and at 2 weeks after PFF surgery, the postoperative FIM. ADL recovery was defined as a return to the preinjury ADL level [18]. ADL levels were distinguished between those requiring independent walking and those requiring human assistance. Independent walking was defined as the ability to walk without human assistance, with or without the use of any aids [19]. ADL level was assessed at the last observation before the fracture, the preinjury ADL, and at 2 weeks and at the last observation after PFF surgery. The independent walk rate was defined as the proportion of patients who were able to achieve independent walking. This was assessed at 1 or 2 weeks postoperatively. The readmission rate was calculated as the percentage of patients who were hospitalized again for any reason after discharge until the final observation period. The reoperation rate was defined as the percentage of patients who required any surgical procedure [20]. In the Beals and Tower classification, the postrevision status was graded into 3 grades according to previous reports: excellent (stable arthroplasty with minimal deformity), good (stable arthroplasty or with minimal subsidence and fracture healed with moderate deformity), or poor (loosening, nonunion, sepsis, severe deformity, or new fracture) [21]. The radiolucent line around the stem was defined as a radiolucent interval of 2 mm or less at the cement-bone or implant-bone interfaces [22]. These were evaluated using radiography at the final observation. Physicians not directly involved in each treatment assessed the outcomes. The primary outcome was defined as the independent walk rate.

### Data analyses

Continuous variables are described as median and ranges, and categorical variables are described as absolute numbers and frequencies. The *Mann-Whitney U* test was used to compare continuous variables between 2 groups, and *Fisher’s exact* test was used to compare categorical variables between 2 groups. Pain VAS scores were compared between the 2 groups for postoperative improvement using a 2-way repeated measures analysis of variance. *Kaplan-Meier* curves were drawn to assess the survival rate, with readmission and death as endpoints. Statistical significance was defined as *P-*values < .05.

## Results

**Figure 3:**
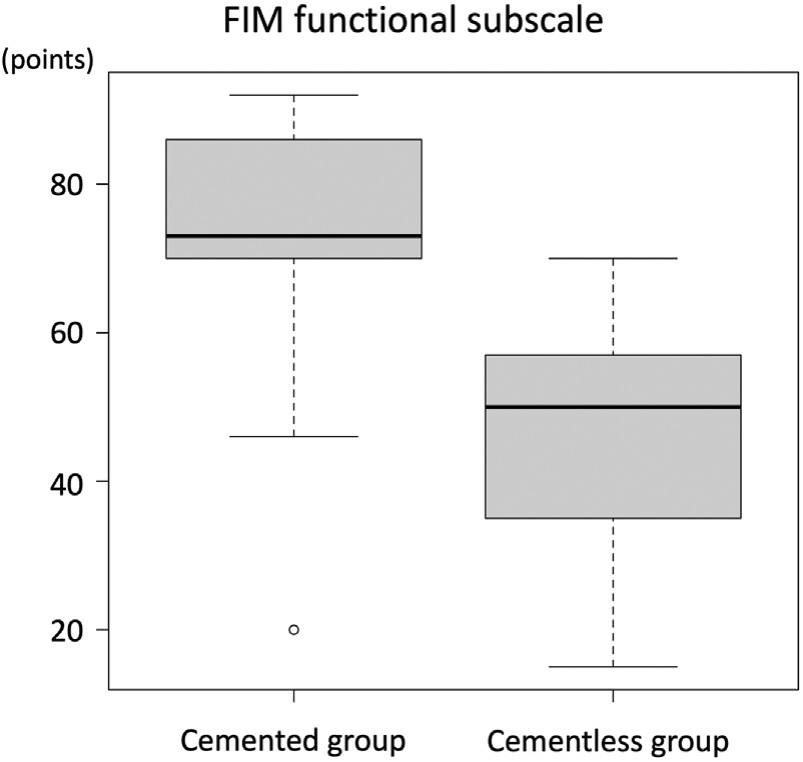
Postoperative functional recovery. Box-and-whisker plots of the FIM functional subscale is shown. The cemented group had significantly better FIM scores than the cementless group.

**Figure 4:**
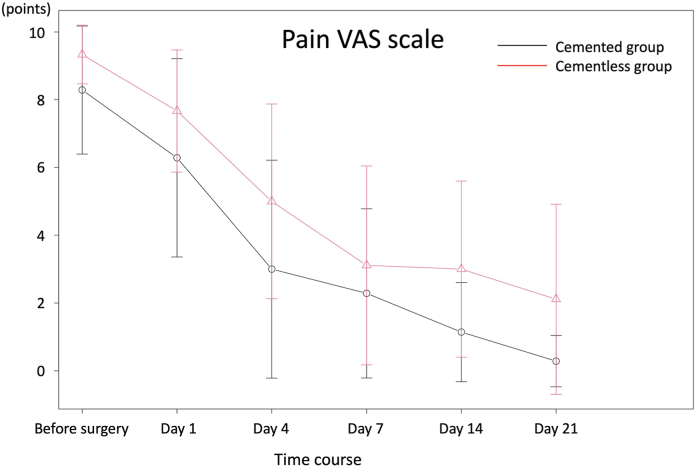
Postoperative pain VAS scale. The postoperative pain trends are shown as a line graph. Pain decreased over time, with no statistically significant differences between the 2 groups.

**Figure 5:**
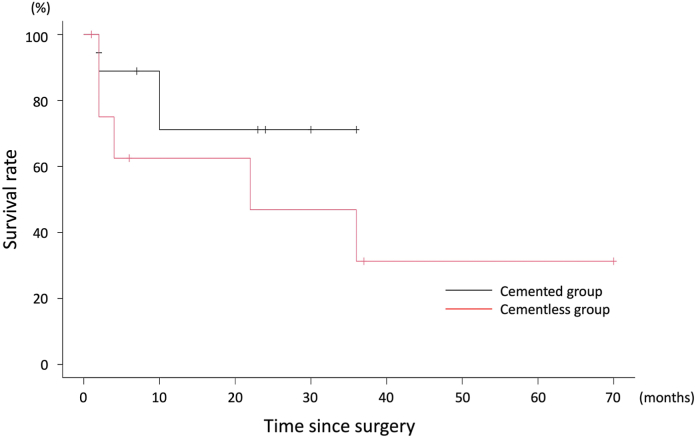
Survival rate of revision surgery. The Kaplan-Meier curve with readmission and death as the endpoint is shown. No statistically significant differences were found between the 2 groups.

The patient demographics are shown in Table 1. The outcomes were summarized in Table 2. The cemented group demonstrated a shorter surgical time (149 vs 273 minutes, *P* = .01), lower transfusion rate (11 vs 89%, *P* < .01), and lower maximum CPK values (681 vs 1064 units/L, *P* = .01) than the cementless group. In addition, patients in the cemented group recovered functional mobility earlier than those in the cementless group, with higher postoperative FIM functional subscale values (73 vs 50 points, *P* = .02) (Fig. 3), a higher independent walk rate (89 vs 11%, *P* < .01), and more ADL recovery at 2 weeks postoperatively (100 vs 44%, *P* = .03). Radiological parameters were similar between the 2 groups, especially cemented stem revision, which did not have a negative impact on bone union if the reduction of the fracture was done precisely. The differences in preoperative and postoperative pain VAS were 6.89 in cemented group and 6.33 in cementless group, both exceeding the minimal clinically important difference. However, the postoperative improvement in pain VAS scores did not differ between the 2 groups (*P* = .11) (Fig. 4). Survival rates were not significantly different between the 2 groups (*P* = .27) (Table 3, Fig. 5). At 2 years postoperatively, there was no difference in the postrevision reoperation rates between the 2 groups. Specifically, only one patient in the cementless group required reoperation for polyethylene insert exchange due to dislocation. There were no cases of reoperation due to nonunion or loosening after revision in this cohort.

**Table 1:** Patient demographics.

Parameters	Cemented group (n = 9)	Cementless group (n = 9)	*P-*values
Age at the operation (y)	81 (54-92)	79 (56-90)	.57
Woman (%)	8 (89)	6 (67)	.58
Body mass index (kg/m^2^)	21.4 (16.4-26.3)	19.9 (13.8-30.6)	.44
ASA physical status (%)			.99
Class Ⅱ	7 (78)	7 (78)	
Class Ⅲ	2 (22)	2 (22)	
Preinjury ADL (%)			.99
Independent walk	8 (89)	9 (100)	
Assistance required	1 (11)	0 (0)	
Initial FIM functional score	40 (13-57)	35 (13-57)	.99
Initial FIM mental score	35 (10-35)	31 (22-35)	.42
Primary THA prostheses (%)			.99
Cementless	1 (11)	0 (0)	
Cemented	8 (89)	9 (100)	
Time since primary THA (mo)	2 (1-756)	13 (1-335)	.82
Follow-up period since revision surgery (mo)	23 (2-36)	32 (1-71)	.35

Median and range were provided. Number of cases and percentage were also provided.

ASA, American Society of Anesthesiologists.

**Table 2:** Outcomes.

Parameters	Cemented group (n = 9)	Cementless group (n = 9)	*P-*values
Operative parameters			
Surgical time (min)	149 (114-365)	273 (194-420)	.01^a^
Intraoperative blood loss (g)	600 (500-1600)	800 (395-1600)	.54
Transfusion rate (case, %)	1 (11%)	8 (89%)	.003^a^
Serum CPK maximum (U/L)	681 (359-1099)	1064 (585-2397)	.01^a^
Serum CRP maximum (mg/dL)	13.2 (5.98-22.63)	11.06 (5.00-15.06)	.44
Serum albumin drop (g/dL)	1.40 (0.80-2.20)	1.80 (1.40-2.30)	.16
Clinical parameters			
Initial Harris hip score	29 (18-70)	32 (23-49)	.69
Postoperative Harris hip score	81 (53-96)	68 (39-93)	.16
Postoperative FIM functional subscale	73 (20-92)	50 (15-70)	.02^a^
Postoperative FIM mental subscale	35 (10-35)	31 (18-35)	.37
ADL recovery at postoperative 2 wk (%)			.03^a^
Recovery to original level	9 (100)	4 (44)	
Assistance required	0 (0)	5 (56)	
ADL recovery at final follow-up (%)			.21
Recovery to original level	9 (100)	6 (67)	
Assistance required	0 (0)	3 (33)	
The independent walk rate at postoperative 1 wk (%)	8 (89)	0 (0)	.001^a^
The independent walk rate at postoperative 2 wks (%)	8 (89)	1 (11)	.003^a^
Readmission rate (case, %)	1 (11)	3 (33)	.58
Reoperation after revision (case, %)	0 (0)	1 (11)	.99
Hospitalization period (d)	35 (18-69)	50 (24-110)	.12
Radiological parameters			
Beals and Towers classification (%)			.81
Excellent	6 (67)	4 (44)	
Good	2 (22)	2 (22)	
Poor	1 (11)	3 (33)	
Radiolucent line (%)	4 (44)	2 (22)	.62

Median and range were provided. Number of cases and percentage were also provided.

CRP, C-reactive protein.

a Denotes statistical significance (*P* < .05).

**Table 3:** Reasons for readmission and survival rates.

Parameter	Cemented group	Cementless group
Reasons of readmission		
Hip fractures on the opposite side	2 (22%)	0 (0%)
Pneumonia or urinary tract infection	1 (11%)	2 (22%)
Dislocation	0 (0%)	1 (11%)
Death	0 (0%)	2 (22%)
Survival rates		
At 2 mo after revision (%)	88.9 (43.3-98.4)	75.0 (31.5-93.1)
At 6 mo after revision (%)	88.9 (43.3-98.4)	62.5 (23.9-86.1)
At 12 mo after revision (%)	71.1 (23.3-92.3)	46.9 (11.9-76.3)
At 24 mo after revision (%)	71.1 (23.3-92.3)	31.2 (9.8-64.1)

Survival rate and 95% confidence interval were provided. End point was defined as readmission after revision for any reason. *Log-rank* test demonstrated that survival rates were not statistically different between 2 groups (*P* = .269).

## Discussion

This study demonstrates that revision using a cemented stem achieved a shorter time to walk independently and earlier ADL recovery than the cementless stem for UCS type B2 PFFs in older patients. When an older patient suffers a lower extremity fracture, muscle weakness and ADL decrease during bed rest [5]. Early postoperative out-of-bed mobilization in older patients who have lower-extremity fractures has been demonstrated to be advantageous for improving ADL independence postoperatively and reducing the cost of care [23]. Specifically, for hip fractures, surgery should be performed as soon as possible to shorten the bed rest period, and early exercise, including walking, is recommended [24]. The importance of early mobilization to prevent disuse and improve ADL should be recognized in PFF, which is common among the older patients as well [4]. This is the first report to compare the usefulness in improving postoperative functional recovery between cemented stem revision and cementless stem revision for PFF UCS type B2.

Because osteointegration is the final fixation between the bone and prosthesis in the cementless stem, excessive micromotion due to load stress concentration in the early postoperative phase may prevent osseointegration in revision using cementless stems [12,25,26]. Therefore, in cementless stems, because the design concept uses biological fixation as the final goal, although cementless stems with current press-fit technology rarely cause problems, it makes sense to have the patient rest postoperatively to avoid excessive micromotion. However, in cemented stems, because the final fixation between the bone and prosthesis can be obtained immediately after surgery using bone cement, loading stress restriction is not necessary in theory [12,25]. However, bone cement may be undesirable for bone union at fracture sites. A previous report focused on nonunion in cases of PFF revision with a cemented stem rather than a cementless stem [27]. However, another study reported that bone union could be achieved with cemented stems if surgeons removed the bone cement from the fracture gap [28]. In our cohort, bone union was achieved in all cases, and nonunion should not be a problem in cemented stem revision as long as the fracture site is reduced accurately. In revision cases with cemented stems in this study, although weight-bearing was not restricted in the immediate postoperative phase, there were no failures, such as nonunion or displacement, in the cemented group during the follow-up period. Furthermore, it is assumed that allowing full weight-bearing load training immediately after surgery in the cemented stem group contributed to the early functional improvement. Therefore, we believe that this procedure is clinically safe and useful, especially in the older patients. The use of cemented stems can also help avoid the risk of intraoperative fractures that are exacerbated when using cementless stems in patients who have poor bone quality, as is often reported in THA or bipolar hip arthroplasty (BHA) for hip fracture [[28], [29], [30]]. Many patients who have PFFs show poor bone quality [4]. Therefore, revision using a cemented stem also has the advantage of decreasing the risk of intraoperative fractures compared with cementless stems.

Cemented stems may also reduce surgical invasiveness. This study demonstrated a shorter surgical time and a lower maximum CPK value in the cemented group. A shorter surgical time for hip arthroplasty is associated with lower risks of mortality [31] and infection [32]. In addition, although there is a wide spread in the data and interpretation should be done with great caution, the low CPK may reflect less muscle damage during the surgical procedure [33]. This could have been influenced by plate use. The longer operative time in the cementless group may be mainly due to the greater exposure of the fracture site to plate fixation. A previous report noted that a larger surgical field was associated with increased surgical invasiveness, increased blood loss, and increased surgical time [34]. Anemia in older patients has been reported to have a negative impact on postoperative functional recovery [35], and cemented THA itself has been reported to reduce perioperative blood loss [36]. These findings demonstrate that cemented stem revision has a positive effect on postoperative functional recovery by avoiding excessive surgical invasiveness.

Since there were no critical failures at 2-year postoperative follow-up, the surgical procedure can be considered safe in clinical settings. Cemented stem revision permitting full weight bearing immediately after surgery did not seem to compromise safety in the mid-term postoperative period. However, a report stating that the use of bone cement is associated with a slight increase in intraoperative mortality discourages surgeons from using cemented stems. Intraoperative mortality increased with cemented BHA compared to cementless BHA in a report from the Norwegian registry (0.3% vs 0.04%, *P* = .02) [37]. Surgeons should be aware of the small but potentially increased risk of intraoperative mortality when using bone cement. However, for simplicity, it has also been noted that patients who underwent BHA with a cemented stem were in poor general condition and not only had poor bone quality [38]. In fact, research removing bias has demonstrated that the mortality risk of cemented stems is not significantly higher than that of cementless stems [39]. Although surgeons should make decisions in each case, it has recently been reported that the use of a cemented stem is clinically useful in hip arthroplasty for patients who have poor bone quality, as typified by the older patients, to gain early functional recovery and pain relief [40].

A limitation of this study is the small number of cases. Therefore, it is possible that potentially important outcomes were not detected due to the low statistical power [41]. However, statistically significant differences in outcomes regarding functional recovery were clearly observed, which were sufficient to reject the null hypothesis in this study design [42]. Clear differences will be observed as the number of cases increases.

## Conclusions

Our study demonstrates that revision using a cemented stem for UCS type B2 fractures in older patients significantly improves early postoperative functional recovery. These findings emphasize the potential benefits of selecting the appropriate stem type in similar cases, contributing to enhanced patient outcomes and postoperative care.

## Acknowledgments

We appreciate the facility's medical staff.

## Conflicts of interest

The authors declare there are no conflicts of interest.

For full disclosure statements refer to https://doi.org/10.1016/j.artd.2024.101467.

## CRediT authorship contribution statement

**Hideki Ueyama:** Writing – review & editing, Writing – original draft, Visualization, Validation, Resources, Methodology, Investigation, Formal analysis, Data curation. **Mitsuyoshi Yamamura:** Supervision, Project administration, Methodology, Investigation, Conceptualization. **Junichiro Koyanagi:** Investigation, Data curation. **Kenji Fukunaga:** Methodology, Investigation. **Susumu Takemura:** Investigation, Data curation. **Suguru Nakamura:** Visualization, Resources, Methodology, Investigation, Data curation, Conceptualization.

## Appendix A Supplementary Data

Conflict of Interest Statement for Fukunaga

Conflict of Interest Statement for Koyanagi

Conflict of Interest Statement for Nakamura

Conflict of Interest Statement for Takemura

Conflict of Interest Statement for Ueyama

Conflict of Interest Statement for Yamamura
