# Supplementary material for: Early Postoperative Functional Recovery in Older Patients With Periprosthetic Femoral Fractures: Comparison Between Cemented and Cementless Stem Revisions
Source: Arthroplast Today. 2024 Jul 20;28:101467. doi: 10.1016/j.artd.2024.101467 (PMC11295462; doi:10.1016/j.artd.2024.101467)
Supplement: Conflict of Interest Statement for Yamamura [file mmc6.pdf]

INDIVIDUAL CONFLICT OF INTEREST STATEMENT

American Association of Hip and Knee Surgeons

(Adopted from the American Academy of Orthopaedic Surgeons disclosure statement)

The following form **must be filled out completely and submitted by each author (example, 6 authors, 6 forms). All items require a response. If there is no relevant disclosure for a given item, enter "*None*."**

Manuscript Title: Early Postoperative Functional Recovery in Older Patients with Periprosthetic Femoral Fractures: Comparison Between Cemented and Cementless Stem Revisions

1. Royalties from a company or supplier (The following conflicts were disclosed) **None**

2. Speakers bureau/paid presentations for a company or supplier (The following conflicts were disclosed) **None**

3A. Paid employee for a company or supplier (The following conflicts were disclosed) **None**

3B. Paid consultant for a company or supplier (The following conflicts were disclosed) **None**

3C. Unpaid consultants for a company or supplier (The following conflicts were disclosed) **None**

4. Stock or stock options in a company or supplier (The following conflicts were disclosed) **None**

5. Research support from a company or supplier as a Principal Investigator (The following conflicts were disclosed)

   None

6. Other financial or material support from a company or supplier (The following conflicts were disclosed) **None**

7. Royalties, financial or material support from publishers (The following conflicts were disclosed) **None**

8. Medical/Orthopaedic publications editorial/governing board (The following conflicts were disclosed) **None**

9. Board member/committee appointments for a society (The following conflicts were disclosed) **None**

Each author must sign AND print or type his/her name, date and submit a separate form

In addition, one BLINDED Conflict of Interest form (no author names used) should be submitted per manuscript with all author disclosures.

Mitsuyoshi Yamamura	Mitsuyoshi Yamamura	Aug / 19 / 2024
Author Name (Print or Type)	Author Signature	Date
